# Strengthening National Disease Surveillance and Response—Haiti, 2010–2015

**DOI:** 10.4269/ajtmh.16-0948

**Published:** 2017-10-18

**Authors:** Stanley Juin, Nicolas Schaad, Donald Lafontant, Gerard A. Joseph, Ezra Barzilay, Jacques Boncy, Robert Barrais, Frantz Jean Louis, Nadia Lapierre Jean Charles, Salomon Corvil, Nickolsno Barthelemy, Amber Dismer, Jean Samuel Pierre, Roodly W. Archer, Mayer Antoine, Barbara Marston, Mark Katz, Patrick Dely, Paul Adrien, David L. Fitter, David Lowrance, Roopal Patel

**Affiliations:** 1 U.S. Centers for Disease Control and Prevention, Port-au-Prince, Haiti;; 2 U.S. Centers for Disease Control and Prevention, Maputo, Mozambique;; 3 Directorate of Epidemiology, Laboratory and Research, Port-au-Prince, Haiti;; 4 National Laboratory of Public Health, Port-au-Prince, Haiti;; 5 U.S. Centers for Disease Control and Prevention, Atlanta, Georgia;; 6 Ben Gurion University of the Negev, Beersheva, Israel

## Abstract

Haiti’s health system has faced many challenges over the years, with competing health priorities in the context of chronic financial and human resource limitations. As a result, the existing notifiable disease surveillance system was unable to provide the most basic epidemiologic data for public health decision-making and action. In the wake of the January 2010 earthquake, the Haitian Ministry of Public Health and Population collaborated with the U.S. Centers for Disease Control and Prevention, the Pan American Health Organization, and other local and international partners to implement a functional national surveillance system. More than 7 years later, it is important to take the opportunity to reflect on progress made on surveillance and response in Haiti, including disease detection, reporting, outbreak investigation, and response. The national epidemiologic surveillance network that started with 51 sites in 2010 has been expanded to 357 sites as of December 2015. Disease outbreaks identified via the surveillance system, or other surveillance approaches, are investigated by epidemiologists trained by the Ministry of Health’s Field Epidemiology Training Program. Other related surveillance modules have been developed on the same model and electronic platform, allowing the country to document the impact of interventions, track progress, and monitor health problems. Sustainability remains the greatest challenge since most of the funding for surveillance come from external sources.

## INTRODUCTION

Emerging pathogens and infectious diseases continue to cause significant morbidity and mortality around the world; increasing global travel and changing environmental conditions compound new developing health threats.^1^ From the Ebola outbreak in West Africa to the Middle East Respiratory Syndrome Coronavirus (MERS-CoV), infectious agents demonstrate the ability to adapt and spread with the potential to affect large and often vulnerable populations. In 2015, the World Health Organization (WHO) recorded more than 150 outbreak reports from 36 countries across all the continents with Zika virus and MERS-CoV as the most commonly reported infections.^2^ With expanding international travel, a local epidemic has the potential to spread quickly across borders.^1^ Thus, it is critical for countries to have well established systems to prevent, detect, and respond to any public health threat.

The International Health Regulations (IHR), revised and adopted in 2005 by the World Health Assembly, were designed to help the international community deal with the risk posed by emerging and reemerging infectious diseases and other health threats.^2–5,6^ As part of their commitment to the IHR, participating countries agreed to comply with these rules by 2012. However, this binding document signed by 196 countries has been difficult to implement as fewer than 20% of countries had met IHR goals.^5–7^ By 2014, only about one-third of participating countries (64 countries) reported fully achieving the core capacities.^8^

The Global Health Security Agenda (GHSA), which includes 55 countries and stakeholder organizations, was launched in 2014 to help nations meet IHR requirements and promote global health security as an international priority.^9^ Priorities under GHSA include assisting countries to develop national infectious disease laboratories, electronic public health reporting systems, emergency operations centers, and effectively trained workforce.^7^ In 2015, Haiti was approved as a Phase 2 GHSA country providing impetus to examine and build Haiti’s disease surveillance systems and preparedness to face new global disease threats.

In January 2010, Haiti’s already fragile health infrastructure was further impacted, after the country experienced a devastating 7.0 magnitude earthquake. Within 2 weeks of the earthquake, the U.S. Centers for Disease Control and Prevention (CDC), the Pan American Health Organization (PAHO) and other national and international agencies began working with Haiti’s Ministry of Public Health and Population (MSPP) to improve disease surveillance in the country.^10,11^ This article will describe Haiti’s progress in disease surveillance, document the lessons learned from the implementation of systems, and inform future investment in public health in Haiti in the context of the GHSA.

## BACKGROUND: DISEASE SURVEILLANCE IN HAITI PRIOR TO 2010

Haiti, an island nation of approximately 11 million people, is geographically divided into 10 departments consisting of 42 arrondissements and a total of 140 communes.^12^ The health-care system follows the same structure, with administrative health units at each level: 1) the Department Health Directorate, 2) the Communal Health Unit now replaced by the Arrondissement Health Unit, and 3) health facility. The country’s disease surveillance systems have generally been passive, and managed by vertical programs. An example of this is the National Tuberculosis (TB) Program, which has conducted TB surveillance since the late 1980s.^12^ TB surveillance is paper based, data are collected by health-care providers, aggregated in monthly reports, and stored at the departmental level. Data validation meetings are organized on a quarterly basis with the departments and the health-care providers.^13–16^ Another example, the human immunodeficiency virus/acquired immune deficiency syndrome (HIV/AIDS) surveillance system, started in 2008,^14^ is facility based and receives reports from all facilities where HIV testing and counseling services are provided. Aggregated and case-based data are entered by a data clerk at the health facility onto a web-based platform for aggregate reporting, or by health-care providers through one of the three electronic medical record systems currently in use across the country.^14,16^ These vertical surveillance systems were created out of a necessity to collect key data in the absence of a broad, national integrated surveillance system that could provide actionable data for all priority diseases.

Disease surveillance has been a functional part of the public hygiene division of MSPP since 1975 when the program commenced to collect epidemiologic reports from health districts on mandatory reportable diseases. Between 1988 and 1991, MSPP implemented a Sentinel Surveillance Network, mainly for vaccine-preventable diseases (VPDs) such as acute flaccid paralysis, measles, neonatal tetanus, and meningococcemia, which paved the way for the expansion of the surveillance system. Initially established in the metropolitan area of the capital, Port-au-Prince, this network was later expanded to cover all departments. It was then managed by a Haitian nongovernmental organization until 1999, when funding from international donors decreased considerably. Operational management of the sentinel surveillance network was transferred to MSPP; however, decreased funding and political turmoil resulted once more in a halt in the expansion of the network.^10^

## THE NATIONAL EPIDEMIOLOGIC SURVEILLANCE NETWORK (NESN)

**Figure 1.**
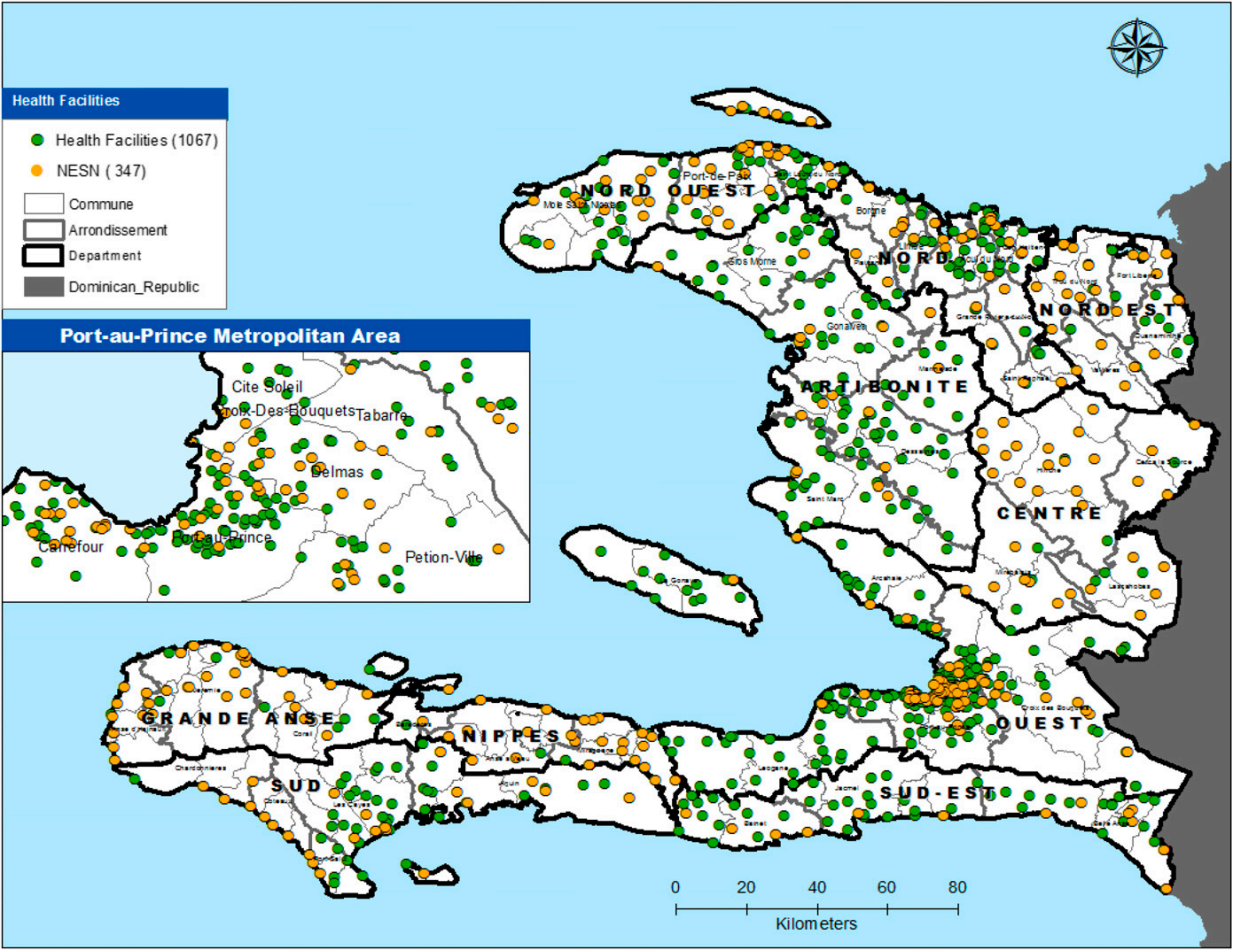
Geographic distribution of NESN surveillance sites, December 31, 2015.

In 2010, in collaboration with CDC, PAHO/WHO, and other international partners, the epidemiology division of MSPP created the National Sentinel Surveillance System, to help detect and respond to outbreaks in the postearthquake context.^10^ In its original form, 51 sites reported data on 25 diseases in all 10 departments in the country.^10^ Since the inception of the surveillance system and later the implementation of the national surveillance strategic plan in 2012, the system has evolved toward an integrated paradigm aimed at achieving comprehensive and expanded surveillance, including a gradual shift toward laboratory-based surveillance. From the original 51 sentinel sites in 2010,^9^ the surveillance system has grown to include 347 sites as of December 31, 2015 (Figure 1). The ministry’s strategic plan for surveillance calls for an eventual expansion to include all 1,048 health facilities across the country by 2018, the integration of a community-based surveillance component, and the extension of the diseases under surveillance to include more noncommunicable diseases.

The successful implementation and expansion have relied on the establishment of an expanded epidemiologic workforce, standardized operating procedures for routine surveillance, and an adaptable reporting system. In each department, specialized epidemiologic surveillance officers send weekly reports on the number of cases for 47 conditions, 14 of which are immediately reportable (Table 1). Case counts are aggregated by sex, age group (< 5 years, ≥ 5 years), and morbidity/mortality status.^10^ New age groups have been included since 2015 to align with MSPP recommendations. Because of the limited availability of laboratory diagnostics at health facilities, the surveillance system is primarily syndromic. The surveillance officers collect aggregated data on cases meeting the case definitions for conditions under surveillance. In 2010, there were 56 officers spread across the country collecting data on 25 conditions; by the end of 2015, there were 168 working at all levels of the national health system collecting data on 47 conditions. Surveillance officers working at health facilities collect and report data from the facility. Communal surveillance officers do the same, but for all health facilities within the commune; they also perform data validation for the commune. At the departmental health directorates, the surveillance officers oversee surveillance within the department under the supervision of a departmental epidemiologist. They also work with a deputy departmental epidemiologist, responsible for VPD surveillance, a statistician, and monitoring and evaluation officers.

**Table 1:** NESN conditions under surveillance, December 2015

Immediately reportable	Weekly reportable		
Acute flaccid paralysis	Acute bloody diarrhea	Leprosy suspect	Typhoid suspect
Acute hemorrhagic fever	Acute respiratory infection	Lymphatic filariasis probable	Violence
Animal bite	Acute watery diarrhea	Malaria confirmed	Zika suspect
Any unusual event	Breast cancer	Malaria suspect	
Cholera probable	Cervix cancer	Malaria tested	
Cholera suspect	Chikungunya suspect	Malnutrition	
Congenital rubella syndrome	Cutaneous anthrax suspect	Motor accident	
Diphtheria probable	Dengue suspect	Neonatal tetanus	
Food poisoning	Diabetes	Other conditions	
Maternal death	Epilepsy	Pertussis suspect	
Measles/rubella suspect	Febrile jaundice syndrome	Prostate cancer	
Meningitis	Fever of unknown origin	Sexually transmitted diseases	
Plague suspect	HIV confirmed	Tetanus	
Vaccine-related event	Human rabies	Tuberculosis positive	
	Hypertension		

HIV = human immunodeficiency virus.

Haiti’s Field Epidemiology Training Program (FETP), established in 2011, has trained over 200 MSPP employees including physicians, nurses, laboratory technicians, computer scientists, pharmacists, veterinarians, and surveillance officers in epidemiologic methods, disease surveillance, and outbreak response. These trainees, working closely with departmental epidemiologists, have been involved in detection and response to over 100 outbreaks such as cholera, vector-borne diseases, and food poisoning since the establishment of the program.

Surveillance data are entered into the platform, allowing direct reporting to the departmental and national level. Data are reviewed, validated, and analyzed at the department level before being sent to the national level for further analysis and review, allowing for more immediate decision-making at a regional level prior to the involvement of national authorities. MSPP has used this basic reporting structure when implementing disease-specific surveillance systems and modules as well, namely the VPD surveillance module and the National Cholera Surveillance System, both of which are covered in more detail later on. Weekly national surveillance meetings are convened to present and discuss routine surveillance data analysis and other surveillance systems, as well as share information pertaining to ongoing existing outbreaks and responses.

Since 2010, more than 15 new conditions including chikungunya, Zika, human rabies, diabetes, and maternal deaths have been added to the list of diseases under surveillance to better reflect the epidemiologic situation in Haiti and account for emerging infections. Additional conditions have been integrated without any major disruption of the system by using existing infrastructure and leveraging the relative flexibility of the web platform to incorporate new data elements. Case definitions are developed for each condition under surveillance with subject matter experts from MSPP and partners, and disseminated to the field. In addition, refresher trainings are held yearly for surveillance officers and physicians.

From 2010 to 2015, the yearly cumulative reported number of cases of all diseases under surveillance has steadily increased from just over 200,000 to more than 1.6 million, reflecting the expansion of sites and conditions as well as completeness of reporting from sites. On average, 60% of reported cases of all diseases are female and 30% are < 5 years of age. Febrile illnesses (e.g., typhoid, malaria, dengue, chikungunya, febrile jaundice syndrome, and fever of unknown origin) regularly represent the most frequently reported syndromes, making up on average 13% of yearly reported cases. Suspected malaria was generally the second most reported febrile condition reported, accounting for 21% of all febrile illness reported in 2015. Acute respiratory infections represented on average 7.5% of all reported cases of disease. Diarrhea represented about 10% of all reported cases in 2010, but since then on average 3% of reported cases are diarrheal patients.

Overall, the implementation and gradual evolution of this system have noticeably improved Haiti’s capacity to conduct real-time surveillance, through an inter-operable, inter-connected electronic reporting system that allows the country to monitor disease trends and health indicators, and improve the early detection of health threats. Despite this, some key gaps remain. For example, work remains to increase coordination with the Ministry of Agriculture to better integrate surveillance for zoonotic diseases and coalesce efforts around an integrated One Health strategy.

## THE VPD MODULE

Timely detection and response to VPDs have always been a challenge. In 2013, the ministry, with the assistance of the Brazilian and Cuban governments through a tri-partite consortium, developed a surveillance strategy to leverage the existing surveillance system to improve the detection cases of VPDs and the response to identified outbreaks. This strategy is based on three pillars: 1) improving case identification, 2) active case finding and contact tracing, 3) monitoring and evaluation of the system. Twelve new assistant epidemiologists were hired to oversee VPD surveillance and work closely with surveillance officers and the departmental epidemiologists. The system and impact as described by Tohme and others in this supplement show that significant progress has been made in meeting performance indicators as required for the ongoing certification of measles, congenital rubella elimination, and polio eradication.

**Figure 2.**
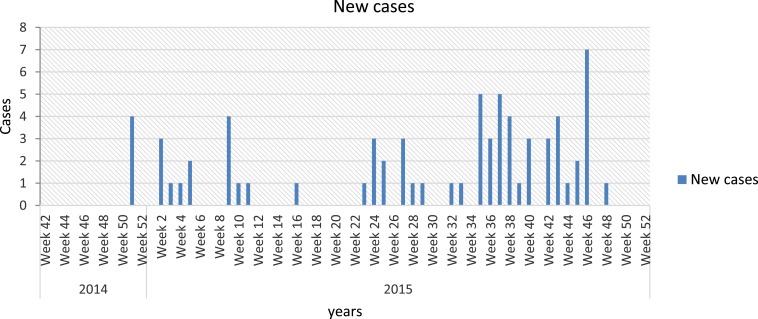
Diphtheria outbreak, October 2014 to December 2015. This figure appears in color at www.ajtmh.org.

As a result of the implementation of the VPD module, Haiti’s capacity to detect VPD outbreaks has visibly improved. In 2014, a cluster of diphtheria cases was initially reported in Port-au-Prince and promptly investigated with a rapid response, which included contact prophylaxis, targeted immunization activities, and enhanced active surveillance in neighboring communes. Enhanced active surveillance quickly identified a much broader outbreak: from Epi week 51 of 2014 to Epi week 48 of 2015, 74 probable diphtheria cases were reported in four of Haiti’s 10 departments (Figure 2). As a part of the case investigations, samples were collected from 40 patients and, of those, 55% (22/40) were laboratory confirmed. The case fatality rate for confirmed cases was 41% (9/22). In response, MSPP and partners including CDC, PAHO, and the United Nations Children’s Emergency Fund developed an integrated strategy to strengthen surveillance, laboratory detection, case management, chemoprophylaxis, and immunization for diphtheria.

## THE NATIONAL CHOLERA SURVEILLANCE SYSTEM (NCSS)

In October 2010, MSPP declared the first ever recorded cholera outbreak in Haiti. This public health event prompted the implementation of the cholera-specific surveillance system to monitor the epidemic and inform the public response. This system was built on the existing routine surveillance infrastructure for data collection, reporting, and data validation. Daily reports of new cases, hospitalized cases, and deaths aggregated by age groups were sent from cholera treatment facilities all over the country.^17^ At the peak of the epidemic, in 2011, there were 254 cholera treatment facilities. Stools samples from across the country were sent to the National Public Health Laboratory in Port-au-Prince for testing.

**Figure 3.**
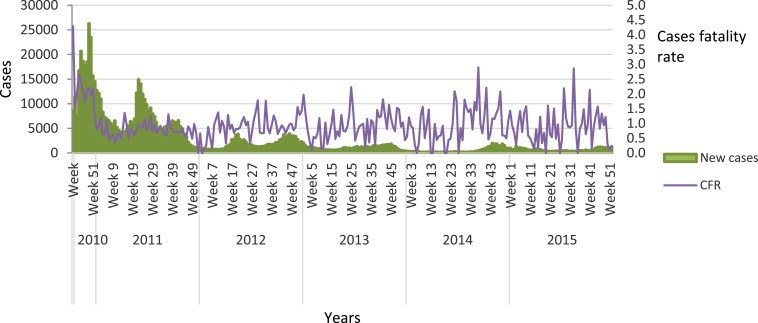
Cholera suspected cases and case fatality rates, October 2010 to December 2015.

From October 2010 to December 2015, MSPP reported a total of 763,842 suspected cholera cases, of which 107,192 (14%) were in children less than 5 years old. In this same period, there were 9,154 deaths with an overall case fatality ratio of 1.2% (Figure 3). Four departments have consistently accounted for most of the cases (West, Artibonite, Center, and North), although flare ups and localized outbreaks continue to be recorded all over the country especially during the rainy seasons (May–June and October–January). Weekly alerts are generated allowing for timely responses from mobile rapid intervention teams. Recent outbreaks, including one in Port-au-Prince, have garnered attention within and beyond the government of Haiti. This has prompted the MSPP to refocus their efforts, coordinate with partners, and examine Haiti’s progress toward cholera elimination. Although the annual number of cases is far fewer than it was initially (36,644 cases in 2015 versus 352,033 cases in 2011), significant challenges remain for controlling the cholera outbreak, including donor fragmentation and lack of sustained funding for long-term investments.

To achieve cholera elimination in Haiti and reduce the burden of diarrheal diseases more generally, strong disease control measures must be implemented and sustained. This includes making significant investments in infrastructure for potable water and sanitation, comprehensive and continuous community sensitization, especially in hotspots, while continuing to evaluate the potential role for oral cholera vaccine in the context of limited global supplies of the vaccine. However, in the face of declining donor funding, additional financial and technical resources are required in both the short and long term to address the staggering challenges facing Haiti’s water and sanitation systems, as well as promote broader global health security priorities.

## LABORATORY-ENHANCED SENTINEL SURVEILLANCE (PRESEPI)

The influx of resources postearthquake and introduction of cholera also helped MSPP in April 2012 to launch laboratory-enhanced surveillance, the first systematic non-HIV/TB laboratory-based surveillance system for infectious diseases in the country.^18,19^ While the national laboratory played a critical role from the onset of the cholera epidemic by identifying *Vibrio cholerae* within a few days of the first case report in each department, providing ongoing culture confirmation of suspect cases, and by performing antimicrobial susceptibility testing essential to developing treatment guidelines, this testing was ultimately inconsistent and nonrepresentative. Laboratory samples for syndromes such as typhoid or malaria were not routinely collected or tested.

Starting in April 2012, MSPP began collecting information on and samples from incident hospitalized cases of diarrhea (acute watery diarrhea and acute bloody diarrhea) and acute febrile illness in four sentinel hospitals.^18,19^ Inclusion criteria for sites included 1) participation in the routine surveillance system, 2) geographic and demographic representativeness, 3) medium to high overall patient volume, and 4) feasible transport of samples to the national laboratory. Subsequently, the laboratory-based surveillance was expanded to three other sites. In 2013, the system was also expanded to include severe acute respiratory infections (SARIs) and suspected meningitis. Surveillance officers are responsible for ensuring that all eligible patients are identified and specimens are collected. They administer a brief demographic questionnaire regarding including symptoms and patient history. Stool samples from diarrhea cases are tested for cholera, *Salmonella*, *Shigella*, as well as rotavirus in the case of patients under the age of 5 years. Blood samples from febrile patients are tested for malaria, dengue, typhoid, and leptospirosis. Cerebrospinal fluid (CSF) samples from meningitis cases are cultured and tested by polymerase chain reaction (PCR) for *Streptococcus pneumoniae*, *Neisseria meningitidis*, and *Haemophilus influenzae*. Nasopharyngeal swabs from SARI cases are tested by PCR for influenza virus type A and B.

**Figure 4.**
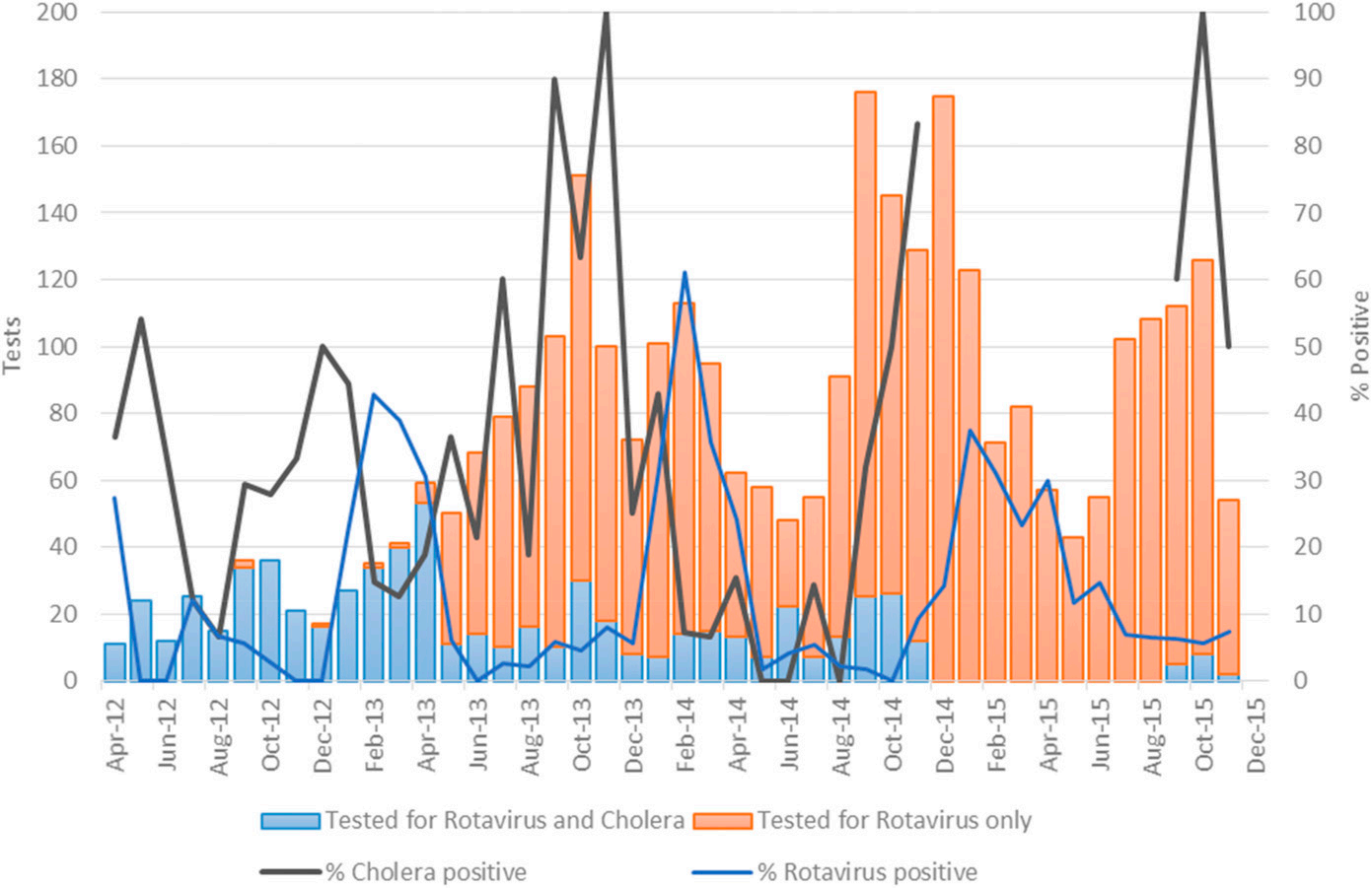
Distribution of cholera and rotavirus positive in under 5 year old, 2012–2015.

**Figure 5.**
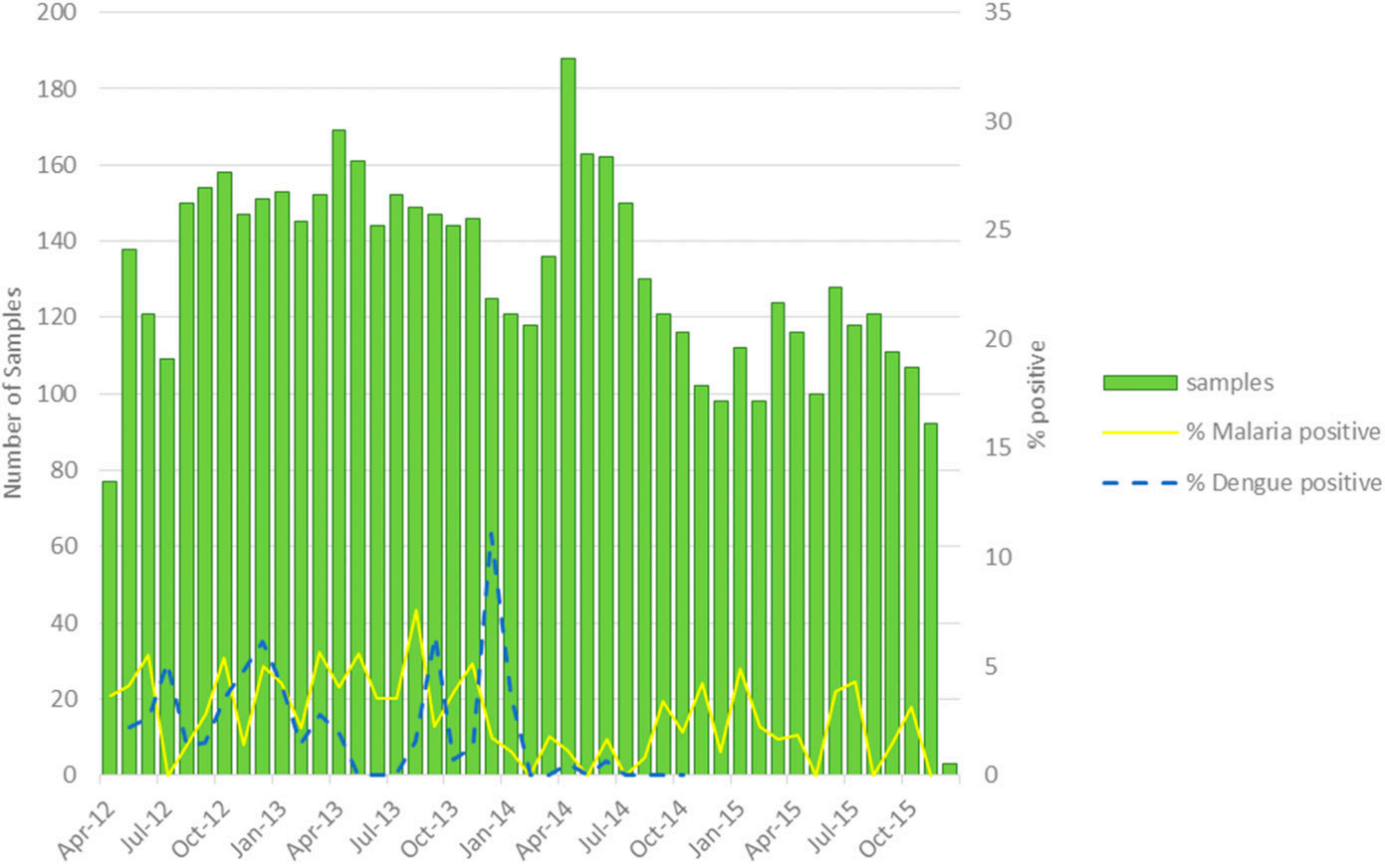
Distribution of malaria and dengue in people greater than 5 years old, 2012–2015. This figure appears in color at www.ajtmh.org.

From April 2012 to December 2015, 12,714 samples were collected: 6,087 stool, 5,827 blood, 661 oropharyngeal, and 139 CSF. Throughout this period, cholera was the main cause of diarrhea among sampled patients 5 years or older and under 5 years old, with 69.3% and 29.0% of samples testing positive by culture, respectively. During this same period, rotavirus was the second most common pathogen in children under 5 with diarrhea (13.9% positive by Premier Rotaclone qualitative EIA) (Figure 4). Although suspect malaria represents about 25% of all febrile illnesses reported to routine surveillance, from 2012 to 2015, only 2.9% of samples from febrile patients tested positive for malaria by rapid diagnostic test (RDT) (Malaria *P. falciparum* test First Response Ag [113FRC25]) (Figure 5). Dengue represented 1.9% of cases of febrile illness reported to NESN, 1.9% samples testing positive for IgM and NS1 by RDT (SD Bioline Dengue Duo Rapid Diagnostic Test) (Table 2).

**Table 2:** Cases reported through NESN, NCSS, PRESEPI, 2010–2015

NESN	2010	2011	2012	2013	2014	2015
Acute respiratory infections	28,019 (13.0%)	49,769 (7.3%)	65,974 (7.5%)	89,886 (8.4%)	112,690 (8.2%)	126,336 (7.5%)
Fever	45,730 (21.3%)	84,364 (12.4%)	116,808 (13.3%)	138,107 (12.8%)	194,601 (14.1%)	162,042 (13.2%)
Diarrhea	21,023 (9.8%)	30,921 (4.5%)	32,421 (3.7%)	37,047 (3.4%)	39,515 (2.9%)	57,632 (3.4%)
Meningitis	0 (0.0%)	64 (0.0%)	83 (0.0%)	189 (0.0%)	292 (0.0%)	314 (0.0%)
Reportable conditions*	50,084 (23.3%)	24,422 (3.6%)	29.735 (3.4%)	38,832 (3.6%)	52,495 (3.8%)	98,718 (5.9%)
Other conditions†	70,056 (32.3%)	493,084 (71.6%)	631,244 (72%)	771,536 (71.7%)	976,348 (70.9%)	1,231,684 (73.4%)
Total	214,912 (100%)	682,624 (100%)	876,265 (100%)	1,075,597 (100%)	1,375,941 (100%)	1,676,726 (100%)

NCSS	2010	2011	2012	2013	2014	2015
Cases seen						
< 5	18,772	47,267	17,960	10,228	5,402	7,642
≥ 5	166,579	304,766	83,543	48,740	23,854	29,492
Total	185,351	352,033	101,503	58,968	29,256	37,134
Hospitalized cases						
< 5	7,715	20,408	9,018	5,515	3,530	5,608
≥ 5	96,013	166,265	52,859	32,470	18,186	24,420
Total	103,728	186,673	61,877	37,985	21,716	30,028
Hospitalized death	2,521	1,950	597	400	304	321
CFR (%)	2.43	1.04	0.96	1.05	0.99	0.74

PRESEPI 2012–2015							
Specimen	Test	Received	Tested	Positive	*Haemophilus influenzae*	*Streptococcus pneumoniae*	*N*eisseria *meningitidis*
CSF	PCR	139	139	26 (18.7%)	11 (7.9%)	15 (10.8%)	0 (0.0%)
Stool	Culture				Cholera	*Shigella*	*Salmonella*
	< 5	3,596	704	214 (30.4%)	202 (29.0%)	9 (1.3%)	3 (1.2%)
	≥ 5	2,423	2,273	1,608 (71.0%)	1,576 (69.3%)	25 (1.1%)	7 (0.3%
	Age unknown	68	51	27 (53.0%)	26 (51.0%)	0 (0.0%)	1 (2.0%)
	Total	6,087	3,028	1849 (61.1%)	1804 (60.0%)	34 (1.1%)	11 (0.4%)
	Rotavirus < 5	3,527	3,182	444 (13.9%)			
Blood		5,827	5,524	499 (9%)	
	Tubex		3,030	268 (11.3%)
	Leptospira		3,073	39 (1.3%)
	Malaria RDT		5,003	143 (2.9%)
	Dengue RDT		3,447	67 (1.9%)

					AH1N1	AH3N2	AH1N9	B
Oropharyngeal	PCR	661	439	36 (8.2%)	12 (2.7%)	10 (2.3%)	0 (0.0%)	14 (3.2%)

CFR = case fatality ratio; CSF = cerebrospinal fluid; NCSS = National Cholera Surveillance System; PCR = polymerase chain reaction; PRESPEI = laboratory-enhanced sentinel surveillance; RDT = rapid diagnostic test.

* Other reportable conditions.

† Other conditions not under surveillance.

Since 2012, data on the etiology and pathogen-specific burden of key infectious disease syndromes have enabled MSPP and other public health policy decision-makers to set evidence-based priorities for optimal use of limited resources for public health programs. These data will be useful for the evaluation of public health interventions such as the introduction of the rotavirus vaccine in 2014, the pneumococcal conjugate vaccine, scheduled for 2018, and possibly the oral cholera vaccine.

## LOOKING FORWARD

Haiti has made incremental progress toward developing a robust, timely surveillance system characterized by collaboration between governmental and nongovernmental institutions at the commune, department, and central levels. Routine surveillance has continuously evolved since 2010, expanding in scale and scope with more sites reporting on more conditions every year since. The ability to add diseases and conditions to the system before and during outbreaks, and if necessary set up enhanced complementary components such as cholera surveillance exemplifies the system’s adaptability, scalability, and flexibility. The planned integration of community-based surveillance will improve detection of health threats in rural communities where health-care utilization is very low.^20^

In line with CDC and WHO guidelines on Integrated Disease Surveillance and Response, the system should be integrated to coordinate and streamline the portfolio of existing surveillance activities and to maximize efficiency and resources, rather than trying to maintain separate vertical activities.^21^ The experience of establishing a surveillance system of national scope in Haiti utilizing existing surveillance infrastructure highlights the fact that it is more efficient to integrate existing disease surveillance data flows into a matrix-like structure where vertical (or in-depth) surveillance systems capture the information necessary to meet the surveillance data needs of a horizontal (or broad) system. Cholera surveillance provides a working example of a vertically designed module capable of being integrated into the horizontal routine system. Such an integrated and centralized platform of surveillance is a step in helping Haiti to conform to the 2005 IHR core capacity requirements for surveillance and response.^22,23^

Through a resolution in 2008, WHO emphasized the role of public health laboratories by calling for the organization of a national public health laboratory that would link national, regional, subregional, and international laboratories.^24^ Internationally, the national laboratory has already developed partnerships with the relevant regional laboratory (Caribbean Public Health Agency) and reference laboratories (e.g., CDC-Atlanta). Within the country, the national laboratory is working on implementing in-country regional laboratories that will greatly improve the laboratory component of the surveillance system and ensure that laboratory results are linked in surveillance system with cases. Integrating laboratory confirmation into routine surveillance and its complementary systems is essential to minimize delays in taking public health action.^20^ The laboratory-enhanced surveillance system represents a significant step in the long-term transition toward laboratory-based surveillance and a model for how the laboratory testing can be best integrated into routine surveillance activities as capacity in this field continues to grow. However, much work remains to be done along this axis. As one example of an area for future improvement, the ongoing implementation of electronic laboratory information management systems will allow testing results to be linked with case data in real time. Furthermore, laboratory diagnostic capacity at the level of health facilities across the country needs to continue to be improved in parallel to the strengthening of regional reference laboratories. To achieve this, in parallel to building diagnostic capacity at this level, sites should be encouraged to routinely send samples to the national laboratory as a part of their quality assurance program.

Reliable surveillance systems also rely on staff capable of reporting, analyzing, disseminating, and responding to surveillance results for appropriate public health action.^20,25^ Eighty percent of Haiti’s FETP graduates continue to work within MSPP, hold leadership positions at the national and departmental level. These graduates and future residents will be crucial to link public health surveillance function and participation across all levels of the health-care system. To continue to grow Haiti’s human resources capacity, Haiti should continue to develop intermediary and frontline personnel such as surveillance officers, who may not be as skilled, but can perform the functions without significant financial burden to the country.^26^

Among all of these developments, it would be unwise to not address the system’s sustainability. Since 1975, MSPP has implemented surveillance systems in the country; however, fluctuating donor funding and a lack of substantial governmental financial investment have always threatened, and sometimes disrupted, the stability of these systems. The routine surveillance and other surveillance systems continue to be financed by outside sources, threatening overall sustainability. Surveillance should continue to be a focus for MSPP in the coming years to prevent the collapse of these achievements; however, without national financial investments in surveillance, uncertainty will continue to persist.

## CONCLUSION

Although one intention of this article was to offer a balanced account that could serve as a roadmap for public health professionals working to implement surveillance activities in Haiti and similar countries across the world, a secondary intention was to highlight how these emergency response efforts can be leveraged in support of long-term health systems strengthening. In a 1959 speech, President John F. Kennedy famously used Chinese calligraphy as an allegory for the potential duality of outcomes following a crisis:

The Chinese use two brush strokes to write the word ‘crisis.’ One brush stroke stands for danger; the other for opportunity. In a crisis, be aware of the danger—but recognize the opportunity

Going forward, the intention and hope is to harness the lessons learned during the response to the Haiti earthquake and cholera outbreak and use the surveillance infrastructure that has been created as a foundation on which to build a robust, integrated disease surveillance system that will carry forward a legacy to address Haiti’s surveillance needs and its future health threats. The GHSA present an opportunity for the country to continue to build on these efforts to achieve the ultimate goal of IHR compliance.
